# Diseases of the Blood

**Published:** 1905-02-04

**Authors:** 


### DISEASES OF THE BLOOD.

(*Continued from page* 318.)

**Splenic Anæmia of Infancy.**—Whatever the functions of the spleen are, they can easily be taken up by other organs, for excision leads to no bad results. It probably acts as a filter in removing foreign particles from the blood-stream. This occurs physiologically in the removal of the débris of red and white corpuscles, and its enlargement in acute infective disease is probably in response to an increased demand for these scavenger functions. The Malpighian bodies certainly form lymphocytes. During intra-uterine life the spleen forms red corpuscles, but this does not persist as a normal function after birth. Most cases of splenic anæmia of infancy occur between the ages of six months and two years. The complexion is sallow; rickets is very often present; congenital syphilis occasionally. The liver is usually somewhat enlarged, whilst the lymphatic glands never attain any great degree of enlargement. Catarrhal complications, such as diarrhœa or bronchitis, are often present and may cause death. In severe cases a petechial eruption may appear and is usually a herald of death. The disease may occur in more than one member of a family, and twins are especially liable to suffer. The red blood cells are always reduced in number, but rarely to an extreme degree, the hæmoglobin is always reduced, but not necessarily in proportion to the reduction of red cells. The red cells are apt to vary a good deal in size, and nucleated forms are often met with. The number of the latter bears no constant relation either to the total number of red cells or the degree of leucocytosis. The number of leucocytes varies; it may actually be diminished, but is more commonly increased; if it is increased, it may affect the granular or the non-granular forms. Polymorphism in the leucocytosis is as marked in this disease as in myelogenous leukæmia. These blood changes are of importance from the point of view of prognosis—the degree of splenic enlargement is of little import, but the lower the number of red cells and the higher the number and the greater the polymorphism of the white cells the worse the outlook. The chief change found in the spleen is a chronic splenitis affecting chiefly the pulp, but sometimes invading the Malpighian bodies; this goes on to the greatest degree of fibrosis in the cases of longest standing. The marrow of the bones shows evidences of great cellular activity. The nature of the disease is very doubtful; some authorities think that it is not a definite disease, but merely a secondary anæmia, which owes its peculiar manifestations to the fact of its occurrence in the early years of life, others that it is merely an aleukæmic stage of a true leukæmia, others consider that it stands halfway between leukæmia and pernicious anæmia, whilst others, again, think that it is a disease *sui generis*. In regard to these various views, it must be admitted that there is more reason to class these cases by themselves clinically than pathologically, for the disease has no pathological anatomy peculiar to itself; the changes in the spleen are the same in kind as are met with in, for instance, rickets and congenital syphilis. The enlargement of the spleen bears no relation to the degree of blood change, and it is difficult to understand how a mere fibrous hyperplasia of the spleen can produce an increased destruction of the blood. The changes in the leucocytes and red marrow may be the consequence of interference with the function of the spleen. In the disease called Banti's disease, or splenic anæmia of adults, although the histological changes in the spleen are very similar, yet the blood picture is totally different from that of splenic anæmia of infancy. In the former there is a slight diminution of red cells, a marked diminution of hæmoglobin and a diminished number of leucocytes. Again, the clinical course is different, the disease in adults is a chronic and progressive one and leads to death, whereas the condition in infancy is very often recovered from. Further, one occasionally meets with cases of the adult type in childhood though not before the second dentition.

(*To be continued.*)

